# Super-Refractory Status Epilepticus (SRSE) Secondary to Neurosyphilis Complicated by a Prolonged Drug-Induced Cardiac Arrest Successfully Bridged With Extracorporeal Membrane Oxygenation (ECMO) and Impella®: A Case Report

**DOI:** 10.7759/cureus.118335

**Published:** 2026-10-21

**Authors:** Daniela Gutfreund, Lucca Loretz, Andreas Bloch, Lara Diem, Sascha Albrecht, Matthias Bossard

**Affiliations:** 1 Department of Intensive Care, Lucerne Cantonal Hospital, Lucerne, CHE; 2 Department of Neurology, Lucerne Cantonal Hospital, Lucerne, CHE; 3 Department of Infectious Diseases, Lucerne Cantonal Hospital, Lucerne, CHE; 4 Department of Cardiology, Lucerne Cantonal Hospital, Lucerne, CHE

**Keywords:** anticonvulsants, cardiac arrest, cardiogenic shock, drug-induced arrhythmias, impella, l-type calcium channel, neurosyphylis, super-refractory status epilepticus (srse), venoarterial extracorporeal membrane oxygenation (va-ecmo), voltage-gated sodium channels (nav)

## Abstract

Neurosyphilis is a rare but readily treatable cause of status epilepticus (SE). Nevertheless, SE has a substantial morbidity and mortality if treatment is refractory and/or life-threatening complications occur. Veno-arterial extracorporeal membrane oxygenation (VA-ECMO) has previously been successfully employed following life- threatening arrhythmias with refractory circulatory failure due to antiseizure medications (ASM) in the context of SE.

Here we describe the combined use of VA-ECMO along with an Impella CP® micro-axial pump to provide full circulatory support and left ventricular unloading in a patient with a presumed drug-induced, refractory cardiac arrest amidst treatment for super-refractory status epilepticus (SRSE). This patient presented with a recurrent episode of convulsive SE with subsequent treatment-refractory nonconvulsive seizure activity on incremental ASM therapy. Investigations consistent with neurosyphilis prompted the initiation of antimicrobial therapy. ASM adjustments were made in view of suspected lacoseamide-induced arrhythmias, but subsequently a treatment-refractory cardiac arrest occurred with ensuing pulseless electrical activity and asystole. Mechanical life support followed by VA-ECMO was promptly initiated and an Impella CP® device was then placed given a non-ejecting heart. Subsequently, hemodynamic stabilization and seizure control were readily achieved and full cardiac recovery occurred after seven days.

VA-ECMO plus Impella® provided effective circulatory support under which seizure control could be achieved and cardiac function recovered. Increased pro-arrhythmic risks due to possible synergistic inhibition of cardiac ionic channels need to be anticipated when multiple ASM and anesthetic agents are introduced during SE treatment. Neurosyphilis needs to always be considered as a rare cause of SE.

## Introduction

Status epilepticus (SE) has substantial morbidity and mortality, influenced by factors such as the underlying cause, the patients' age and the duration of the convulsive or non-convulsive SE (NCSE) [1]. Refractory SE (RSE) is characterized by persistent or recurrent seizure activity that does not respond to first- and second-line antiepileptic treatment or requiring general anesthesia. An estimated 10%-15% of hospital-admitted cases with SE will progress to a super-refractory status epilepticus (SRSE), defined as ongoing seizure activity beyond 24 h despite treatment with anesthetic agents or recurrence during anesthetic wean [2,3]. SRSE has a poor prognosis, especially once significant neuronal destruction has occurred. Hence, prompt initiation of anti-epileptic treatment, identification of underlying treatable causes and expedient interdisciplinary management is crucial to prevent or limit irreversible neural injury and mortality [1].

The risk of antiseizure-medication (ASM)-induced arrhythmias is low for healthy individuals on a single agent [4,5]. However, during SE, multiple ASM and anesthetic agents are employed, which may target multiple cardiac ionic channels, such as the main cardiac voltage-gated sodium channel (NAV1.5), the L-type calcium channel and the human ether-à-go-related gene (hERG) potassium channel. This may result in synergistic pharmacodynamic interactions, thereby increasing the risk of life-threatening arrhythmias and cardiac arrest.

Venoarterial extracorporeal membrane oxygenation (VA-ECMO) provides full respiratory and circulatory support, in which blood derived from a large vein is oxygenated and returned by a centrifugal pump to the central arterial system, either via cannula directly placed in the aorta (central VA-ECMO) or via cannulation of a peripheral, i.e. axillary or a femoral artery (peripheral VA-ECMO) [6]. The latter is easier to initiate without sternotomy, typically achieving flow rates of 3-6 L/min. VA-ECMO increases left ventricular (LV) afterload impeding LV outflow and, therefore, LV venting strategies are often required to prevent LV distention, thrombus formation and pulmonary edema. VA-ECMO has been successfully used in severe poisoning and drug- and toxin-induced cardiogenic shock and cardiac arrest to bridge to cardiac recovery with good outcomes in selected patients [7-11]. The American Heart Association 2025 Guidelines recommend early VA-ECMO initiation for refractory cardiogenic shock or lethal arrhythmias in acute drug intoxication once standard treatment fails [12]. Furthermore, several reports have described the use of VA-ECMO for cardiogenic shock induced by ASM in the setting of SE with good neurological and overall outcomes [13-17].

Impella® (Abiomed, Danvers, MA, USA) is a catheter-mounted microaxial flow pump system that is placed into the left ventricular (LV) cavity, providing antegrade blood flow to the ascending aorta, and when used combined with VA-ECMO is referred to as ECMELLA or ECPELLA (these are two different fusions of the acronyms of ECMO and IMPELLA) [18]. Impella CP® is inserted percutaneously and can provide average flow rates between 3.0 and 4.0 L/min. In patients with refractory cardiac arrest or cardiogenic shock, ECPELLA has been shown to effectively provide LV unloading and to augment circulatory volume. Observational studies and recent meta-analyses have shown a survival benefit in patients with cardiac arrest treated with ECPELLA compared to ECMO alone [19, 20]. Here, we report a case of a refractory cardiac arrest during SRSE treatment, rescued with ECPELLA.

## Case presentation

A 49-year-old Eastern European male migrant worker with a six-month history of new-onset epilepsy presented with repetitive generalized tonic-clonic seizures on treatment with valproic acid. Prehospital management included midazolam and levetiracetam application. The initial Glasgow Coma Scale (GCS) was six upon arrival in the emergency department. The initial electroencephalography (EEG) revealed NCSE, which only briefly responded to clonazepam administration. The patient was transferred to the ICU for further management.

Initial diagnostic assessment

Magnetic resonance imaging (MRI) showed marked cerebral frontal atrophy and hemosiderin deposition. Cerebrospinal fluid (CSF) analysis revealed lymphocytic pleocytosis. Neurosyphilis was suspected and subsequently confirmed by Treponema Pallidum serology in serum and CSF (Table 1). Cross-sectional imaging by computed tomography (CT) of the thorax and abdomen revealed no evidence of syphilitic gummas, aortitis, or aneurysms. A transthoracic echocardiogram (TTE) was unrevealing and there was no evidence of ocular involvement on a designated ophthalmological exam.

**Table 1:** Selected initial laboratory investigations Abbreviations: Ab, antibody; CSF, cerebrospinal fluid; HIV, human immunodeficiency virus; IgG, immunoglobulin G; IgM, immunoglobulin M; MTBC, Mycobacterium tuberculosis complex; PCR, polymerase chain reaction; QFN, QuantiFERON;  RSV, respiratory syncytial virus; RT-PCR, reverse transcription polymerase chain reaction; SARS-Cov2, severe acute respiratory syndrome coronavirus 2; TBEV, tick-borne encephalitis virus; Tp, Treponema pallidium; TPHA, Treponema pallidum hemagglutination assay; VDRL, Venereal Disease Research Laboratory

Test	Result
Tp Ab (total)	Positive
VDRL (Serum)	Reactive, 1:64
TPHA	Positive, 1:≥40960
Tp immunoblot (IgG)	+++
VDRL (CSF)	Reactive, 1:16
TPHA (CSF)	Positive, 1:20480
HIV-1/2 Ab	Negative
HIV-1 p24 Ag	Negative
*Borrelia burgdorferi* Ab (IgG)	Negative
*Borrelia burgdorferi* Ab (IgM)	Negative
Influenza A RT-PCR	Negative
Influenza B RT-PCR	Negative
SARS-CoV-2 RT-PCR	Negative
RSV RT-PCR	Negative
TBEV Ab (IgG)	Negative
TBEV Ab (IgM)	Negative
QFN MTBC-specific Ag	Negative
Valproic acid TR (347-693 µmol/L)	334 µmol/L
Blood alcohol concentration	<0.008%

Therapeutic intervention

Upon ICU admission, he received an intravenous (iv) loading dose of valproic acid (2400 mg), followed by 800 mg every 12h, in addition to levetiracetam (1500 mg every 12h) (Figure 1). However, despite first- and second-line ASM therapy, NCSE persisted on continued EEG monitoring. Concerns regarding airway protection necessitated endotracheal intubation prior to the initiation of burst suppression with propofol and midazolam.

**Figure 1:**
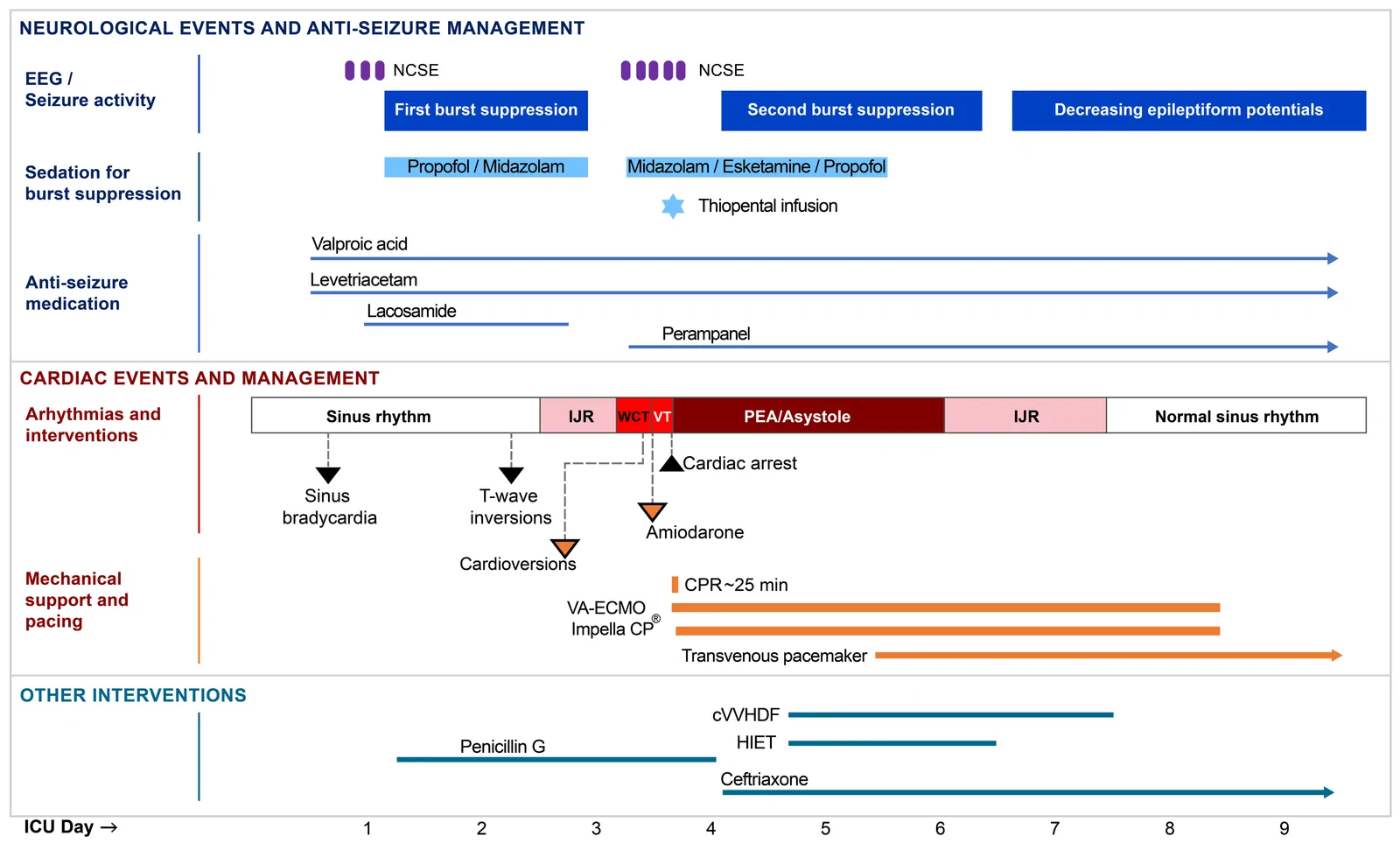
Timeline of neurologic and cardiac events, and therapeutic interventions following ICU admission, days 1-9. Upper panel illustrates neurologic events and anti-convulsive treatment, the mid panel depicts cardiac events and their management and in the lower panel selected other interventions are shown. Abbreviations: NCSE, Nonconvulsive status epilepticus; IJR, intermittent junctional rhythm;  WCT, wide complex tachycardia; VT, ventricular tachycardia; PEA, pulseless electrical activity; cVVHDF, continuous venovenous hemodiafiltration; HIET, high-dose insulin euglycemic therapy.

An ECG obtained on admission day (D) 1 showed sinus bradycardia (Figure 2a). He was hemodynamically stable on minimal vasopressor support. Burst suppression was achieved with propofol, and lacosamide (iv loading dose 400 mg, followed by 200 mg every 12 h) was introduced as adjunctive therapy and high-dose penicillin G (iv) was initiated for treatment of neuro-syphilis (Figure 1). On D2, the ECG showed a borderline QTc prolongation of 508 ms and anterolateral T-wave inversions (Figure 2b). There was no change in hemodynamics. By the end of day D2, the patient required increasing vasopressor support accompanied by intermittent junctional escape rhythms with increasing T-wave inversions and QTc prolongation to 520 ms (Figure 2c). There were no electrolyte abnormalities explaining the ECG changes (Table 2) and therefore lacosamide was discontinued given the suspicion of lacosamide-induced conduction disturbances. On D3, Methylprednisone (125 mg iv x 3 days) was initiated given hemodynamic instability and concerns of a possible Jarisch-Herxheimer reaction. Following a partial reduction of the sedation, continuous EEG monitoring revealed NCSE reoccurrence, prompting the initiation of perampanel (24 mg loading dose, followed by 12 mg once daily) and reinstatement of burst-suppression therapy using incremental midazolam infusion and the addition of esketamine (50 mg). However, adequate burst suppression could not be achieved (Figure 1). Therefore, thiopental sodium infusion (375 mg) was initiated, which induced burst suppression but was immediately followed by the onset of wide complex tachycardia (Figure 2d). Following several unsuccessful attempts at electrical cardioversion, the administration of amiodarone 300mg (iv) led to conversion into a narrow complex tachycardia with an intermittent grade III AV block.

**Table 2:** Selected circulatory support interventions and investigations, days 1-10 Daily maximum ranges where available or daily single values are shown for QTc, troponin, lactate, Mg and K with reference ranges provided in brackets. Abbreviations: LV, left ventricular; Absent, no detectable left ventricular contraction on echocardiography; SB, sinus bradycardia; NSR, normal sinus rhythm; IJR intermittent junctional escape rhythm; WCT, wide complex tachycardia; PEA, pulseless electrical activity; PR, paced rhythm; D/C, discontinued; NE, not evaluated.

Parameter	Day 1	Day 2	Day 3	Day 4	Day 5	Day 6	Day 7	Day 8	Day 10
LV systolic function	Normal	NE	Absent	Absent	Absent	Severely reduced	Mildly reduced	NE	Normal
ECPELLA			+	+	+	+	+	+	D/C
Pacing					+	+	+	+	D/C
Rhythm	SB	NSR, IJR	WCT, PEA, Asystole	Asystole	Asystole, PR	IJR, PR	IJR, NSR, PR	NSR, PR	NSR
QTc (ms)	423	508	520					415	NE
Lactate (0.5-1.6 mmol/l)	0,9	1,2	1,6-15	1,4-1,8	2,7-5,6	3,1-4,3	2,3-3,4	1,1	NE
Troponin (<14 ng/l)	ND	8	27-150	33-66	25-32	NE	31	NE	NE
Mg (0.66-1.07 mmol/l)	0,8	1,01	1,11	0,8	0,66	0,63	0,82	0,81	0,72
K (3.4-4.5 mmol/l)	3,8	3,4-3,7	3,1-5,5	3,4-4,9	4,2-4,6	3,7-4,2	3,7	5,1	4,2
Creatinine (59-104 umol/l)	104	104	NE	81	70	61	69	69	51

Cardiac arrest occurred despite increasing vasopressor administration. Hence, mechanical cardiopulmonary resuscitation was commenced and after several cycles of amiodarone and epinephrin administration, pulseless electrical activity (PEA) with normocardic broad-complex QRS and periods of asystole ensued. Given the refractory cardiac arrest, peripheral VA-ECMO support was initiated via right femoral, arterial and venous access. In view of severe LV impairment without demonstrable ejection on TTE (Table 2), an Impella CP® device was subsequently inserted via the left femoral artery. A coronary angiogram done at that time showed no obstructive coronary artery disease. The patient stabilized under these measures and markers of systemic hypoperfusion markedly improved (Table 2). On D4, continuous veno-venous hemodiafiltration (CvvHDF) was initiated to facilitate drug clearance, and hyperinsulinemic euglycemic therapy (HIET), adapted from beta-blocker toxicity protocols, was commenced. Furthermore, the antibiotic therapy was switched from benzylpenicillin to ceftriaxone, and metronidazole was added for suspected aspiration pneumonia. On D5, a provisional transvenous pacemaker was implanted in view of ongoing asystole with regular isolated p-waves (Figure 2e). A repeat TTE showed no contraction of the LV and severely diminished right ventricular (RV)- function. On D6/7, a junctional rhythm and then normal sinus rhythm returned, and increasing LV systolic function was seen on TTE (Table 2) with the return of a physiological blood pressure amplitude on day 6. After a course of levosimendan-loading VA-ECMO and Impella CP® support was successfully weaned and sequentially removed on D8 after five days of mechanical support. The patient subsequently remained in a stable sinus rhythm (Figure 2f) and the TTE demonstrated normal LV and RV function on D10.

**Figure 2:**
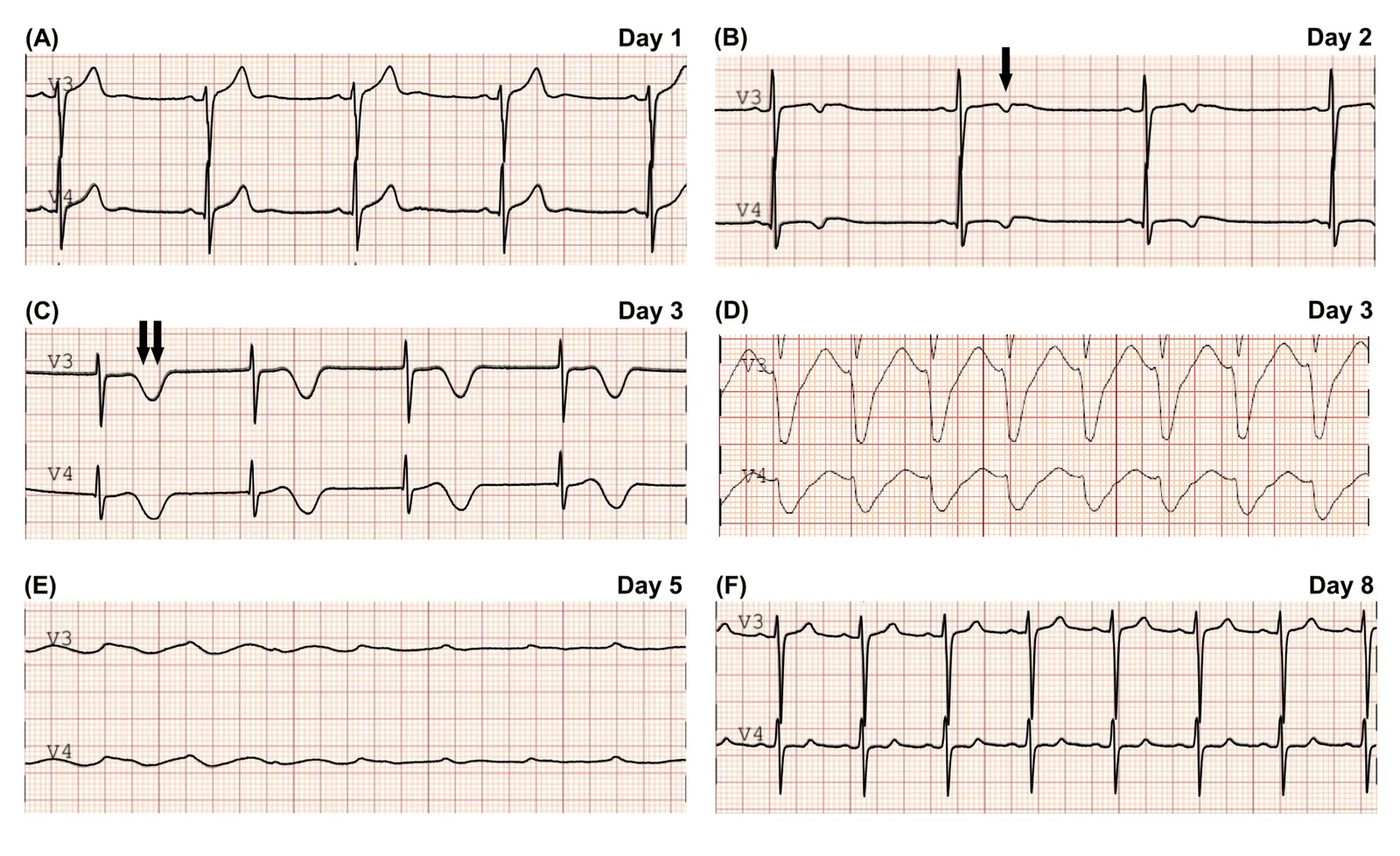
Dynamic ECG changes following ICU admission Selected ECG leads V3 and V4 are shown (paper speed: 25 mm/s; voltage calibration: 10 mm/mV). (A) Sinus bradycardia on day 1. (B) T- wave inversions in V3-V4 (↓) and QTc prolongation (508 ms) on day 2. (C) Intermittent junctional escape rhythms with progressive T- wave inversions  (↓↓)  and QTc prolongation (520 ms) on day 3. (D) Wide complex tachycardia on day 3. (E) P-wave asystole prior pacing under ECMO and Impella® support on day 5. (F) Stable sinus rhythm with normal QTc- Interval (415 ms) on day 8. QT intervals were measured manually, and QTc values were calculated using Bazett’s formula (QTc=QT/√RR with QT and RR expressed in seconds).

Further course and follow-up

After further stabilization, sedation was reduced and EEG monitoring demonstrated improving background activity under intensified antiseizure therapy with the addition of gabapentin and clonazepam. Respiratory recovery was prolonged, requiring tracheostomy, and severe oropharyngeal dysphagia with silent aspiration necessitated PEG placement. The patient gradually regained alertness and partial orientation, though mobility remained therapy-dependent. After 28 days in the ICU, he was transferred to the neurology ward, where agitation and delirium were managed with quetiapine and risperidone. No seizure-suspect events occurred on antiseizure therapy with brivaracetam, valproate, gabapentin and clonazepam. After a further two weeks on the neurology ward, he could be transferred to an inpatient neuro-rehabilitation facility. Dysphagia required continued PEG feeding, but the tracheostomy tube could eventually be removed. Perampanel was successfully tapered under EEG monitoring, and follow-up EEG showed improved background activity without epileptiform discharges. The patient demonstrated marked functional recovery, including independent ambulation, autonomous self-care, and significant cognitive improvement allowing discontinuation of reorientation training. He was repatriated to his home country for continued neurorehabilitation four months after his initial admission.

Further attempts post-admission to obtain a detailed collateral history from his wife eventually revealed that prior to coming to Switzerland, he had been working in another European country, in which he had been hospitalized six months prior with a new diagnosis of SE and syphilis for three days after which he was repatriated to his home country, but treatment was not completed.

## Discussion

To the best of our knowledge, this is a first report describing the use of ECPELLA to rescue a prolonged cardiac arrest amidst treatment for SRSE. The observed arrhythmias and ensuing cardiac arrest were presumably precipitated by the various ASM and anesthetic agents employed for seizure control. There was no preexisting heart disease. Stress (Takotsubo) cardiomyopathy appeared unlikely in view of a globally depressed LV function without areas of hyperkinetic myocardium and electromechanical dissociation shortly after administration of a known cardioplegic agent [21]. However, cardiac arrest syndrome following the prolonged CPR may have contributed to cardiac dysfunction and the delay in neurologic recovery [22].

VA-ECMO can preserve systemic and end-organ perfusion but raises LV afterload, often leading to LV and left atrial distension, thereby increasing the risk of pulmonary edema and thrombus formation due to stasis [23-26]. Impella® not only enhances circulatory support but also provides efficient LV venting [18,27,28]. Although controversy persists regarding the selection of patients that benefit from ECPELLA, several observational studies and recent meta-analyses have shown a survival benefit and an improved neurological outcome in subsets of patients with refractory cardiac arrest treated with ECPELLA compared to VA-ECMO alone [19,20,29,30]. While data from RTCs are lacking, the potential benefits of a combined circulatory support has to be balanced with the increased risks of bleeding, vascular complications, hemolysis, sepsis and renal failure observed in patients treated with ECPELLA compared to ECMO alone in some of these studies [27,28]. In our patient, given the presence of a non-ejecting heart following established VA-ECMO support, ECPELLA appeared to be the best option.

Our patient had been on several ASMs known to exert inhibitory function on several cardiac ionic channels, thereby likely increasing pro-arrhythmic risks. Lacosamide inhibits Nav1.5 by selectively enhancing its slow inactivation pathway [31,32]. Conduction abnormalities and arrhythmias observed in our patient have been described previously for lacosamide [13,33]. In a retrospective analysis of 17 patients with arrhythmias while on lacosamide, ventricular tachycardia (VT) was the most commonly observed arrhythmia, followed by atrial fibrillation, Mobitz type 1 and type 3 heart block, sinus pauses, QRS widening, and PEA/asystole occurred in a patient with a lacosamide overdose [33]. In our patient, lacoseamide had been discontinued 30 hours before the cardiac arrest; however, lacoseamide may still have contributed to the subsequent conduction abnormalities and cardiac arrest via its effect on NAV1.5 given the serum half-life of 13-16 h [34].

Our patient also had received a thiopental bolus infusion, which was immediately followed by the cardiac arrest. Thiopental sodium is known to cause direct myocardial depression, bradycardia, disruption of autonomic control and peripheral vasodilation, further compromising cardiovascular stability [35]. Thiopental sodium and sodium pentobarbital can block Nav1.5 fast inward sodium current (/_Na_) in high concentrations, leading to negative dromotropy [36-38]. Furthermore, thiopental sodium reduces intracellular calcium availability in cardiac myocytes through the inhibition of L-type calcium channels and interferes with calcium release of sarcoplasmatic reticulum, ultimately leading to a negative inotropic effect [38-40]. Reports on pentobarbital-induced arrhythmias are scarce, but transient bradycardia transitioning into ventricular escape rhythms have been described in a patient with SE [41]. However, this patient was on valproic acid and phenytoin, both known to inhibit Nav1.5. Furthermore, although hypotension is a common side effect of pentobarbital, there are only a few reports on severe myocardial dysfunction/stunning following pentobarbital infusion [16,17]. In both of these reports, patients were also treated for SE and required VA-ECMO to bridge to myocardial recovery. One of these patients was on multiple ASM, including midazolam, diazepam, phenytoin, lacoseamide and propofol [16], whereas the other patient was only on diazepam and midazolam prior thiopental infusion [17]. Both midazolam and diazepam have been shown to also exert negative ionotropic effects via L-type calcium channels [42]. Hence, based on the latter report, midazolam and pentobarbital might possibly have been able to trigger cardiac arrest.

In addition, our patient was also on valproic acid, which is known to inhibit Nav1.5 [43] and propofol that has been shown to inhibit both Nav1.5 [44] and the L-type calcium channel [45]. Although reports on valproic acid-induced arrhythmias are scarce, bradycardia and atrioventricular conduction blocks with toxic and therapeutic levels have been reported [46-48]. Apart from a wide spectrum of arrhythmias related to propofol infusion syndrome [49], propofol has been implicated to cause VT, ventricular fibrillation (VF) and Brugada-like patterns on ECG [50]. Though propofol may shorten or lengthen QTc and its effect on QTc is highly variable, it may have contributed to the QTc prolongation observed in our patient.

Furthermore, our patient was also on levetiracetam, esketamine and perampanel, which are all associated with a low pro-arrhythmic risk. Levetiracetam-induced arrhythmias are uncommon and QTc interval prolongation was not seen in clinical studies [51,52], although QT interval prolongations developing into torsade de pointes have been reported along with electrolyte abnormalities [53] and long QT type 2 syndrome [54]. Perampanel and esketamine have not been shown to be associated with conduction abnormalities or arrhythmias at therapeutic doses, but in the context of a perampanel overdose, bradycardia has been observed [55]. Nevertheless, esketamine, perampanel, propofol and pentobarbital all exert varying degrees of inhibition on the hERG potassium channel, associated with QT prolongation and torsade de pointes. Hence, though the risk of arrhythmias is low for individual drugs at therapeutic concentrations, polytherapy targeting multiple cardiac ionic channels may pose a significantly elevated risk.

Neurosyphilis remains a challenging diagnosis due to its highly varied clinical manifestations, which may occur decades after the initial infection without apparent classic primary or secondary signs [56]. Neurosyphilis has re-emerged as a relevant differential diagnosis, particularly in patients presenting with unexplained encephalopathy or atypical neuroimaging findings, such as cortical atrophy or hemorrhagic signs; and both SE and NCSE are rare, underrecognized presentations [57-59]. Compliance with treatment and an extended follow-up at six, 12 and 24 months for latent or tertiary syphilis is critically important, as illustrated in our patient [60]. Standard therapy for neurosyphilis was started with intravenous penicillin G and then switched to ceftriaxone, which has excellent central nervous system (CNS) penetration [61] and a reduced seizure risk compared to penicillins and certain beta-lactams [62,63]. Furthermore, following the start of treatment with penicillin, a possible Jarisch-Herxheimer reaction was considered in view of the hemodynamic instability, though this is difficult to discern from other causes in a critically ill patient [64]. Case reports have described worsening neurological symptoms, including seizures in patients with suspected Jarisch-Herxheimer reaction [65,66]. We elected to provide a short course of steroids in this patient with neurosyphilis, although supportive evidence is based on case reports and supporting evidence from controlled trials is lacking [60,66-69].

## Conclusions

This case illustrates the successful bridging of a prolonged cardiac arrest encountered during SRSE with the swift initiation of CPR and mechanical circulatory support with VA-ECMO plus Impella® given the presence of a non-ejecting heart. The introduction of multiple ASMs and anesthetic agents that inhibit cardiac ionic channels should be done with caution under continuous cardiac monitoring. Further studies of outcomes in clinical trials of VA-ECMO compared to ECPELLA are needed, as are investigations pertaining to the role of pharmacodynamic antiepileptic drug interactions on pro-arrhythmogenic risks. This case also underscores the importance of overcoming sociocultural and language barriers because of which our patient may have compromised compliance with treatment and follow-up, leading to readmission with SE, a life-threatening complication of neurosyphilis.
